# The Role of Cytokines and Inflammatory Cells in Perinatal Brain Injury

**DOI:** 10.1155/2012/561494

**Published:** 2012-03-11

**Authors:** Ryan M. McAdams, Sandra E. Juul

**Affiliations:** Division of Neonatology, Department of Pediatrics, University of Washington, Seattle, WA 98195-6320, USA

## Abstract

Perinatal brain injury frequently complicates preterm birth and leads to significant long-term morbidity. Cytokines and inflammatory cells are mediators in the common pathways associated with perinatal brain injury induced by a variety of insults, such as hypoxic-ischemic injury, reperfusion injury, toxin-mediated injury, and infection. This paper examines our current knowledge regarding cytokine-related perinatal brain injury and specifically discusses strategies for attenuating cytokine-mediated brain damage.

## 1. Introduction

Preterm birth affects 12.5% of pregnancies in the United States [1, 2] and is the leading cause of neonatal morbidity and mortality, accounting for nearly half of the long-term neurologic morbidity in children [3]. The majority of premature infants in developed countries survive; however, 5–10% of survivors develop cerebral palsy (CP), and 40–50% develop cognitive and behavioral deficits [4, 5]. The prolonged vulnerability of the developing white and gray matter to excitotoxic, oxidative, and inflammatory forms of injury is a major factor in the pathogenesis of perinatal brain injury. While acute catastrophic brain injuries sometime occur (e.g., severe intraparenchymal hemorrhage), injury to white and gray matter regions is most often the cumulative result of metabolic, infectious and/or inflammatory, and hypoxic-ischemic insults over time [6]. For example, early respiratory compromise and systemic hypotension can precipitate glutamate, free radical, and cytokine toxicity to developing oligodendrocytes and neurons. The clinical course might be further complicated by late-onset or necrotizing enterocolitis (NEC). These sequential events result in different topographic patterns of injury based on developmental and genetic susceptibilities.

Although there has been much focus on white matter injury (WMI) in premature infants, gray matter abnormalities in cortical and deep nuclear structures, and cerebellar injuries are also common and likely contribute to development of cognitive delay, psychomotor delay, and CP [7]. A variety of inciting events such as hypoxic-ischemia, infection, and/or inflammation, can stimulate a cascade of secondary responses, including fluid-electrolyte imbalance, regional blood flow alterations, calcium-mediated cellular injury, free-radical generation, oxidative and nitrosative stress, glutamate-induced excitotoxicity, disturbances in proinflammatory cytokine production, mitochondrion function, and apoptotic cell death [6, 8]. These disturbances result in activation of inflammatory cells involved in the innate immune response including neutrophils, macrophages, and resident microglia, which may propagate brain injury through mechanisms that directly and indirectly lead to neuronal and preoligodendrocyte (preOL) cell death or dysfunction.

Cytokines and inflammatory cells are mediators in the common pathways associated with perinatal brain injury induced by a variety of insults [9–12]. A better understanding of the role of cytokines in perinatal brain injury is needed to facilitate the development of strategies to prevent and/or treat cerebral white and gray matter damage.

## 2. Cytokines Affecting the Fetus and Neonate: What Are They and Where Do They Come From?

Cytokines are small, cell signaling nonstructural proteins involved in regulating hematopoiesis, inflammation, and immune cell proliferation and differentiation. They are grouped into different classes based on biological activity [13]. The term cytokine encompasses a variety of soluble proteins including monokines, interleukins (IL), colony-stimulating factors, interferons (IFNs), tumor necrosis factor (TNF), and chemokines [14]. These messenger molecules link the neural, endocrine, and immune systems [15]. Cytokines can be pro- or anti-inflammatory, neuroprotective or destructive, depending on their state and concentration [16]. Although nearly all nucleated cells produce cytokines, they are mainly produced by glial cells in the central nervous system (CNS) or by immune cells, such as helper T cells and macrophages [14]. Stimuli inducing cytokine production may originate remote to, or within the CNS. The origin of cytokines acting within the CNS may include blood-borne and native CNS sources, including immune cells, brain endothelial cells, astrocytes, microglia, and neurons [17–19]. Cytokines act by binding to specific cell surface receptors, which then induce intracellular signaling mechanisms that up- or downregulate transcription factors, leading to pro- or anti-inflammatory reactions. Cytokines with generally proinflammatory properties include TNF-*α*, INF-*γ*, IL-1, IL-6, and IL-18, while cytokines that antagonize the proinflammatory responses include IL-1 receptor antagonist, IL-4, IL-6, IL-10, IL-11, and IL-13, and transforming growth factor (TGF)-*β*. Soluble receptors for proinflammatory cytokines can have similar function. Note that IL-6 appears in both categories.

## 3. Differences in Neonatal and Adult Immune Responses

 The immune system of the fetus and newborn reflects the unique interaction between the developing individual and its host-mother. The developing fetus must avoid precipitating a maternal immune response that results in rejection or preterm delivery, but still must protect itself from intrauterine infection and prepare for the transition from the sterile intrauterine environment to the extrauterine environment that is rich with antigenic challenges. This combination of factors results in a neonatal immune system that differs significantly from its adult counterpart. In comparison to adults, the neonatal immune response is biased towards a Th2 response, with a muted Th1 response [20]. Stimulated neonatal mononuclear cells secrete markedly less of the pro-inflammatory Th1-polarizing cytokines, TNF-*α* and IFN-*γ*, whereas secretion of IL-6, a cytokine with anti-inflammatory and Th2-polarizing properties, is actually greater in neonates than adults. This response is mediated by adenosine, an endogenous purine metabolite with immune-modulatory properties [21–23].

## 4. Barriers to Accessing the Brain

**Figure 1:**
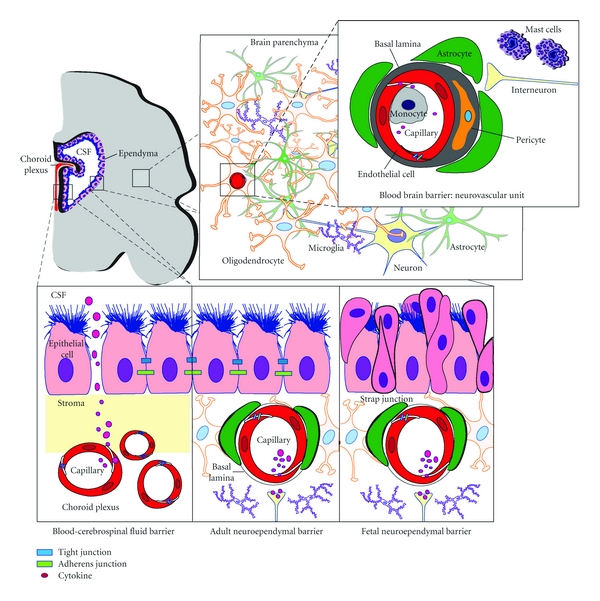
The blood-brain and the blood-cerebrospinal fluid barriers. A schematic diagram of the two barriers that represent the largest interface between blood and brain extracellular fluids: the brain endothelium forming the blood-brain barrier (BBB), also referred to as the neurovascular unit, and the choroid plexus epithelium forming the blood-cerebrospinal fluid (CSF) barrier. The neuroependymal surface lining of the ventricular system (inner CSF-brain barrier) is unique to the fetal brain and is not present in the adult. The neuroependymal cells are connected by “strap junctions” that prevent exchange of large molecules such as proteins between the CSF and brain [31]. Tight junctions and adherens junctions limit paracellular pathway endothelium and epithelium permeability. The neurovascular unit consists of specialized endothelial cells interconnected by tight junctions surrounded by basal lamina in which pericytes are embedded, with an outer ensheathment of astrocytic perivascular endfeet. Mast cells are located at perivascular locations in apposition with astrocytic and neuronal processes [27]. Inflammation may result in disruption of tight junctions and adherens junctions leading to paracellular passage of cytokines.

**Figure 2:**
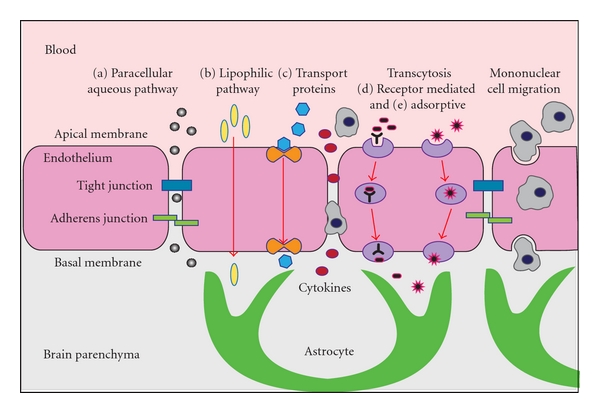
Access pathways across the cerebrovascular endothelial cells. An illustration depicting purposed access routes of materials across the endothelial cells of the blood-brain barrier (BBB). The pathways for cellular molecular movement from the circulation across the BBB may include (a) paracellular aqueous pathway across tight junctions, (b) transcellular pathways including the lipophilic pathway, (c) transport proteins, (d) receptor-mediated transcytosis, and (e) adsorptive transcytosis. Cytokine trafficking may occur via receptor-mediated transcytosis or possibly across disrupted tight junctions in the setting of inflammation. Cytokine movement is thought to occur mainly in the blood-to-brain direction; however, in the blood-cerebrospinal fluid barrier, bulk flow movement may lead to cytokine absorbtion into blood [19]. Mononuclear cells may penetrate the BBB by a process of transcellular diapedesis, directly through the cytoplasm of the endothelial cells without tight junction disruption [29]. During proinflammatory conditions, tight junctions between endothelial cells may be disrupted allowing mononuclear cells to gain access from the blood to the brain via paracellular routes, along with cytokines [30].

There are three interfaces where molecular and cellular exchange between blood and neural tissues or the cerebral spinal fluid occurs. These are the blood brain barrier (BBB) formed by the cerebrovascular endothelial cells between blood and brain interstitial fluid, the choroid plexus epithelium between blood and ventricular CSF (blood-CSF barrier, BCSFB) and the arachnoid epithelium between blood and subarachnoid CSF [24, 25]. The two barriers that represent the largest interface between blood and brain extracellular fluids are the BBB, formed by brain endothelial cells, and the BCSFB, formed by choroid plexus epithelial cells (Figure 1) [26]. The BBB, also termed the “neurovascular unit,” consists of highly specialized endothelial cells interconnected by an elaborate network of complex tight junctions surrounded by basal lamina in which pericytes and perivascular antigen-presenting cells are embedded, with an outer ensheathment of astrocytic perivascular endfeet. Mast cells, which synthesize and store neuroactive and vasoactive substances, are located at perivascular locations on the brain side of the BBB in apposition with astrocytic and neuronal processes [27]. In addition to tight junctions, adherens junctions hold the endothelial cells together providing structural support required for formation of tight junctions and are necessary to prevent disruption of the BBB [26]. The astrocytes that surround the microvasculature provide the cellular link to the neurons and play an active role in signal transduction pathways and regulating the BBB [24]. In adults, there are five known routes by which materials can pass between the circulation and the brain across these barriers (Figure 2) [25]. These are via a paracellular aqueous pathway (across tight junctions) and through transcellular pathways including the lipophilic pathway, via transport proteins, receptor-mediated transcytosis, or adsorptive transcytosis [25, 28]. Whether these same mechanisms are active in the fetus and neonate remains unknown.

From the earliest stages of brain development, the BBB excludes the passage of protein and small lipid insoluble markers between the circulating blood and the brain extracellular fluid [32, 33]. Similarly, paracellular diffusion of protein and small, lipid-insoluble molecules is limited at the BCSFB by apical tight junctions between the choroid plexus epithelial cells [34]. However, these substances may pass by transcellular mechanisms in choroid plexus epithelial cells, and their permeability is much higher in immature compared to adult brain [35]. Stolp et al. studied BBB permeability resulting from lipopolysaccharide-(LPS-) induced systemic inflammation (defined as increased blood concentrations of acute-phase proteins or IL-1*β* and TNF-*α*) in rats and opossums [33]. They demonstrated a restricted period in brain development when protein permeability of the BBB, but not the BCSFB, is altered following systemic inflammation. This increased BBB permeability was specific to white matter and was related to stage of development and not BBB immaturity.

The BBB is a dynamic structure which can be modified by circulating factors or by chemicals secreted by cells associated with the BBB [25]. Agents known to impair adult BBB function (increase leakiness) include bradykinin, histamine, serotonin, glutamate, purine nucleotides (ATP, ADP, AMP), adenosine, platelet-activating factor, phospholipase A2, arachidonic acid, prostaglandins, leukotrienes, interleukins (IL-1*α*, IL-1*β*, IL-6), TNF*α*, macrophage-inhibitory proteins MIP1 and MIP2, free radicals, and nitric oxide (NO) [25]. Many of these agents are upregulated after hypoxia or during infection.

It is not surprising then that localized or systemic inflammation/cytokinemia (e.g., chorioamnionitis and/or fetal inflammatory response) remote to the CNS may result in disruption of the BBB/BCSFB with increased cytokine access to the CNS [36, 37]. Activated CD4+ T lymphocytes, macrophages and dendritic cells must cross the endothelial and the parenchymal basement membranes and glia limitans before gaining direct access to the brain. Transmigration of these cytokine-producing immune cells appears to be influenced by ultrastructural alterations in the laminin isoform composition of the endothelial basement membrane, and by focal matrix metalloproteinase (MMP) activity of the parenchymal basement membrane [24]. To breach to BCSFB, circulating cytokines/immune cells must migrate across the fenestrated choroid plexus capillaries, enter the outer CNS parenchyma, and then penetrate the choroid plexus epithelial cell layer either by passing through the parallel tight junctions strands or transcellularly through the choroid plexus epithelial cells. However, evidence of inflammatory mediator access to the CNS across the BCSFB in the human fetus/neonate remains undefined.

The role of the neurovascular unit, which includes cellular (endothelial and epithelial cells, astrocytes, and pericytes) and acellular (e.g., the extracellular matrix networks) barriers in regulating cytokine access beyond the BBB and BCSFB to the CNS needs to be clarified in order to understand potential opportunities to mitigate the inflammatory cascade associated with perinatal brain injury. There is a paucity of information on *in vivo* human fetal/neonatal properties of barrier dysfunction and the available *in vitro* and adult animal models may not accurately reflect neurovascular unit functional permeability following injury/inflammation. For example, although experimental studies have demonstrated that LPS can induce WMI and neuroinflammation [38], evidence that LPS gains access to the fetal/neonatal brain causing human perinatal brain damage is lacking. However, since microglial cells possess LPS-binding toll-like receptor (TLR)4 receptors and seem to be necessary for LPS-induced oligodendrocyte death [39] this suggests that LPS can gain access to the brain. Additionally, how proinflammatory cytokines affect cellular inward and outward CNS barrier transfer mechanisms and alter CNS barrier function potentially influencing perinatal brain injury remains unknown. Identifying periods when the fetal/neonatal CNS is vulnerable to inflammatory mediator-induced barrier disruption and subsequent damage due to CNS penetration of peripheral toxic molecules is needed in order to define pharmacologic therapeutic windows to access injured brain regions.

The BBB can act as a regulatory conductor between the CNS and the peripheral circulation, establishing and maintaining CNS homeostasis, moderating the nutritional needs of the CNS, and governing influx and efflux of signaling molecules [19]. The BBB appears to have a dual role in regulating immune cell trafficking between the CNS and blood by controlling restrictive and selective permeability [40]. Cytokines can disrupt the BBB [41, 42] and BCSFB, [43] and also can alter saturable neuropeptide transporter [44] and ATP-driven drug efflux pump activity [45] without affecting BBB integrity. The BBB can secrete cytokines [46–49] and may actively participate in inflammatory reactions of the CNS. Dysfunction of BBB and BCSFB mechanisms may be more than just a consequence of inflammation/injury, but also may constitute part of the disease process. Increased blood-spinal barrier permeability following spinal cord trauma involves an active upregulation in inflammatory cytokine transport systems in endothelial cells around the injured area [50]. Immune mediator traffic regulated by the BBB may also play a role in recovery following injury, as has been demonstrated in a murine model of hypothermic brain injury in which macrophages promote early posttraumatic reformation of the BBB [51]. The type and amount of cytokines transported across the BBB varies by CNS region, implying that there are different cytokine-specific regulatory mechanisms and effects [19]. Whether the human fetal/neonatal BBB also plays an active role (similar to animal models) not only in ongoing tissue damage, but also in the recovery process following CNS injury is not clear.

## 5. Infection

An *in utero* infection such as chorioamnionitis may trigger an innate immune system response, resulting in elevated cytokine levels. Microorganisms express conserved sequences known as pathogen-associated molecular patterns (PAMPs), such as LPS and double stranded RNA, on their surfaces. Recognition of these PAMPs by pattern recognition receptors on immune cells stimulate specific host cell TLRs [20]. For example, when stimulated by LPS, TLR4 signals through the adapter molecule myeloid differentiation factor 88, to activate the nuclear factor-*κ*B (NF-*κ*B) pathway that leads to an immune response characterized by the production of cytokines, antimicrobial products, and the regulation of costimulatory molecules [52]. The cytokine response may progress from the trophoblast, decidua, and amniotic epithelium [53, 54], to the amniotic fluid [55, 56] to the fetal lungs and then blood stream, or by direct hematogenous spread via the maternal-placental-fetal circulation. Initiation of a proinflammatory cytokine response following bacterial infection of placental tissues can lead to preterm labor [57]. Cytokines associated with preterm labor include IL-1*β* [58], IL-6 [59], IL-8 [60], and TNF-*α* [61]. Activated immune cells including circulating neutrophils, phagocytic macrophages, T cells, and NK cells, and resident CNS astrocytes and microglia produce biological mediators including cytokines, chemokines, adhesion molecules, and growth factors involved in complex intermolecular interactions that participate in the immunoinflammatory processes related to brain injury [62]. Cytokines in the fetal blood stream may contribute to a systemic fetal inflammatory response with eventual penetration across the BBB resulting in a chemical and or pathogen promoted inflammatory cascade in fetal brain [12].

## 6. Cytokines Expressed by Astrocytes and Microglia

Interaction between the CNS and the immune system relies on the expression of several cytokines and their receptors in both neurons and glial cells in the brain [63]. The two major reactive glial cell types that play significant roles during CNS injury and repair are microglia and astrocytes. These glial cells are involved in the intracerebral immune response where they act, in part, by secreting cytokines, chemokines, neurotrophic, or neurotoxic factors [64]. Cytokines and their receptors, like IL-1*β* and IL-1*β* receptor protein, are constitutively expressed in the CNS by astroglia, microglia, and oligodendrocyte progenitor cells (OPCs) [65].

Astrocytes are important players in neuroinflammatory processes and are capable of producing numerous cytokines including a variety of interleukins, TNF-*α*, and members of the interferon family [66]. The involvement of astrocytes in the pathogenesis of WMI is suggested by increased cytokine expression (IL-1*β*, IL-6, and TNF-*α*) in both the diffuse and focal components of periventricular leukomalacia (PVL) [67, 68]. Activated microglia produce cytokines, chemokines, free radical species, proteases, and other potential mediators of injury [69, 70]. Upon stimulation by LPS, microglia express IL-1*β*, which triggers astrocyte expression of tissue inhibitors of metalloproteinases (TIMPS) [71]. During CNS injury and repair, TIMPS play a critical role in regulating tissue proteolysis by neutralizing the effect of the MMP. TIMP-1 is involved in regulating the growth and morphology of cortical neurons in an MMP-dependent manner [72] and plays a role in oligodendrocyte generation and differentiation [73, 74]. Further studies are needed to determine the role of microglial IL-1*β* cytokine signaling and TIMP expression in perinatal brain inflammation and repair.

## 7. Brain Injury Associated with Prenatal Infection and/or Inflammatory Insults

Intrauterine infection might account for 25–40% of preterm births with up to 80% of preterm deliveries at <30 weeks of gestation having evidence of infection [75]. Clinical chorioamnionitis is significantly associated with cystic PVL and CP [76]. Neonates exposed to clinical chorioamnionitis or histological chorioamnionitis have increased risks of 140% and 80% for developing CP, respectively [77]. Bacterial infection of the decidua and placental membranes activates TLRs on the surface of inflammatory cells which results in release of proinflammatory cytokines, and initiates a local inflammatory reaction in the placenta [78, 79]. Elevated IL-6 concentrations measured in cord blood from neonates with white matter lesions associated with PVL supports the role of intrauterine inflammation and subsequent WMI [80]. Perinatal brain injury may not be contingent on pathogen penetration into the fetal CNS: intrauterine exposure to a systemic inflammatory stimulus alone can lead to brain damage in preterm neonates [10, 81].

Chorioamnionitis can be classified into acute and chronic chorioamnionitis [82]. Acute chorioamnionitis of infectious origin is associated with elevated amniotic fluid IL-6 levels and results from microbial invasion of the amniotic cavity and intrauterine infection. Chronic chorioamnionitis of immunological origin is associated with elevated amniotic fluid CXCL10 levels and is a possible consequence of disrupted immune system hormones affecting CD8+ T-cell activity resulting in maternal antifetal rejection. Amniotic fluid proteomic analysis has demonstrated that acute chorioamnionitis and chronic chorioamnionitis are likely manifestations of different pathological processes [82]. Whether acute versus chronic chorioamnionitis also result in distinct alterations in perinatal brain injury patterns is not known.

## 8. Brain Injury Associated with Postnatal Infection and/or Inflammatory Insults

In preterm infants, known inflammatory conditions are associated with WMI. These include both early- [83] and late-onset sepsis [84], as well as NEC [85] and are generally associated with high plasma levels of IL-6, IL-8, and TNF-*α* [86]. Bronchopulmonary dysplasia, another comorbidity of prematurity, is associated with evidence of inflammation (neutrophils, macrophages, cytokines and toxic oxygen radicals) [87] and is also associated with increased risk of WMI [88].

## 9. Cytokines and Cerebral Palsy

CP, the most common cause of severe physical disability in childhood [89], is an umbrella term describing multiple diseases originating early in life characterized by variable motor impairments secondary to unspecified etiologies and cerebral pathologies. Preterm birth, perinatal infection, and neonatal encephalopathy are important risk factors for the development of CP [90].

### 9.1. Preterm Infants

 Periventricular WMI is an important cause of disability in preterm low-birth-weight infants. Prior to 32 weeks of gestation, preOLs are particularly vulnerable to injury and developmental arrest [91]. Injury to these cells can result in a cystic necrosis of white matter tracts and/or diffuse noncystic lesions with hypomyelination [6]. Injury most commonly occurs in a watershed, periventricular distribution, which typically corresponds clinically with spastic diplegia, the most common form of CP diagnosed in preterm infants [92, 93]. Inflammation, mediated by proinflammatory cytokines, can contribute to the WMI that occurs in preterm infants [94]. In a study of 96 preterm babies with gestational age ≤32 weeks, elevated umbilical cord blood IL-8 concentrations were associated with CP (diagnosed by followup at 1 year of age) [95]. Another large multicenter study of infants with birth weights ≤1000 g (*n* = 1067) demonstrated that circulating IL-8 concentrations were higher on days 0–4 and subsequently in infants who developed CP compared with infants who did not develop CP in both unadjusted and adjusted analyses [96].

Macrophage infiltration and high levels of TNF-*α* and IL-1*β* have been demonstrated in brains of neonates with PVL compared to neonates with anoxic lesions who died shortly after birth [67]. These high cytokine concentrations may have direct cytotoxic effects on oligodendrocytes [97]. Neuronal cytotoxicity following exposure of preOLs to LPS is mediated by activated microglia via TLR-associated signaling pathways [98]. Both focal and diffuse forms of PVL are associated with activated microglia [8]. The release of proinflammatory cytokines from activated microglia has been implicated in neuronal and glia cell death [99]. Pang et al., using primary OPC cultures prepared from neonatal rat optic nerves, demonstrated that LPS-activated microglia mediate OPC death by two distinct mechanisms in a time-dependent manner [100]. An early phase of OPC damage occurs within 24 h after LPS treatment, mediated by NO-dependent oxidative damage, and a delayed phase of OPC death, evident at 48 h after LPS treatment, is mediated by cytokines and is prevented by blocking TNF-*α* activity. Whether these two distinct mechanisms of injury occur in human perinatal brain injury leading to PVL is not clear.

Inflammatory processes originating during vulnerable periods of neurodevelopment may result in perinatal programming. The effects of inflammation triggered by proinflammatory cytokines, prostaglandins, or LPS on the developing CNS of premature infants may have long-term consequences for the individual's ability to cope with environmental exposures during childhood and adulthood [36]. Lin et al. demonstrated that school-age preterm children with PVL-induced CP had significantly higher plasma concentrations of TNF-*α*, increased TNF-*α* released from LPS-stimulated peripheral blood mononuclear cells (PBMCs), and mRNA expression of inflammatory signaling molecules, including TLR4 and TNF-*α*, in PBMCs compared to normal control school-age preterm children [101]. Additionally, intracellular PBMC TNF-*α* levels were significantly higher in children with CP, but lower in controls following LPS stimulation. Whether or not children with CP who were born preterm with a history of PVL have long-term abnormalities of their immune responses remains unclear.

### 9.2. Cytokines, CP, and Neonatal Encephalopathy

Maternal inflammation contributes significantly to fetal susceptibility to hypoxia-ischemia [102–104] and the subsequent development of CP [105, 106]. Hypoxia-ischemia and infection can both induce a systemic inflammatory response associated with elevated cytokines [94, 107]. Higher concentrations of IL-1*β*, IL-6, TNF-*α*, and IL-8 in the blood of neonates who have encephalopathy have been associated with increased anaerobic brain metabolism, and with abnormal neurodevelopmental outcome [108]. Elevated concentrations of IL-6 and IL-8 have been demonstrated in the CSF of asphyxiated full-term infants, with intrathecal levels of these cytokines corresponding to the degree of hypoxic-ischemic encephalopathy [109].

Term neonates with encephalopathy have a risk for CP that is 100 times that of those infants who do not have encephalopathy [10]. Increased concentrations of IL-1*β*, IL-6, and TNF-*α* in amniotic fluid [98], and IL-6 in cord blood [74, 110] secondary to maternal, placental, or fetal infections [97, 111] are associated with cerebral WMI and/or CP. Similarly, elevated neonatal blood concentrations of IL-6 and IL-8 were associated with the diagnosis of CP at 1 year of age in a study of 73 term babies (gestational age ≥36 weeks) [9].

Although infection and/or inflammation increase the risk for CP, they may not be sufficient causal factors to induce brain damage. In a 3-year follow-up study of high-risk infants, Yoon et al. reported that CP was diagnosed in only 18% (5/28) of infants born with documented microbial invasion of the amniotic cavity and 24% (11/45) of infants with evidence of intrauterine inflammation [112]. Another study compared early blood concentrations of inflammatory cytokines (IL-1, -6, and -8 and TNF-*α*) from 64 children later diagnosed with CP to 107 control children (all born at <32 weeks gestational age). Early cytokine concentrations were not predictive of later CP [104].

## 10. Dual-Role Cytokines

Inflammation in the CNS can result in significant brain damage, including injury to axons and myelin, the loss of preOLs, oligodendrocytes, and neurons [69]. However, neuroinflammation can be also be beneficial, promoting neuroprotection, the mobilization of neural precursors for repair, remyelination, and even axonal regeneration [69]. Some cytokines can have both pro- and anti-inflammatory effects. For example IL-4, IL-10, and IL-13 are potent activators of B lymphocytes, and also potent anti-inflammatory agents with the ability to suppress expression of proinflammatory cytokines IL-1 and TNF [13]. TNF-*α* and IL-1*β* can have both neuroprotective and damaging effects [113]. IL-6 and IL-8, typically associated with inflammation, have been associated with the release of nerve growth factor in the CSF of patients with traumatic brain injury suggesting their role in promoting repair of the CNS lesions as well as of axonal regeneration [16]. A dual role can also be seen in macrophages, which are key mediators of the immune response, particularly regarding their ability to produce cytokines. Macrophages can be subdivided into subtypes (M1 and M2) with M1 macrophages considered proinflammatory, producing molecules such as TNF-*α*, IL-1, IL-6, and NO, while the M2 subset is typically considered anti-inflammatory, producing molecules like IL-10, TGF-*β*, and IL-1 receptor antagonist [69]. The neuroimmune response appears to be dichotomous with the balance of pro- and anti-inflammatory cytokines likely influencing neurodevelopmental outcomes. Further research is needed to clarify what influences cytokines (e.g., timing, type, location, and duration of injury) to promote peace or wage war with regard to neuroprotection and neuroinflammation, respectively.

## 11. Cytokines and Genetic Susceptibility to Perinatal Brain Injury

Susceptibility to perinatal brain injury may be partially genetically determined by the balance of proinflammatory and anti-inflammatory cytokine expression. Single-nucleotide polymorphisms in genes encoding cytokines and their receptors might positively or negatively affect the risk of perinatal brain injury in infants. An increased risk for WMI has been associated with IL-8, IL-6, TNF-*α*, and TLR4 polymorphisms [10, 114].

A recent meta-analysis by Wu et al. demonstrated that CP is associated with IL-6 genetic polymorphisms [115]. Moderately preterm infant (32–36 weeks' gestational age) carriers of IL-6 gene −174 C allele, associated with upregulated IL-6 expression, may have an increased risk of developing quadriplegic CP [114]. Functional polymorphism in the IL-6 gene (−174 CC genotype) among term and near-term infants has been associated with an attributable risk percentage of 11.6% for developing CP [116]. The development of hemiplegic and quadriplegic CP has been demonstrated with IL-6 or IL-4 polymorphisms in the presence of viral exposure suggesting an association between candidate cytokine polymorphisms and a fetal inflammatory environment, which may be causally linked to the risk of CP development [114]. This proposed “double jeopardy” hypothesis linking neurotropic viral exposure and genetic susceptibility to infection needs further confirmation in susceptible neonatal population studies to establish causation of CP. In contrast, there may be protective gene polymorphisms. For example, preterm infants (<32 weeks gestation) homozygous for the high IL-10 producer −1082 G allele are significantly less likely to develop ultrasound defined PVL [117].

## 12. Cytokine Biomarkers of Perinatal Brain Injury

Accurate diagnostic, predictive, and prognostic biomarkers of brain injury are needed for optimizing the clinical treatment of at-risk neonates. Ideal biomarkers would accurately reflect the degree of brain injury, the timing and evolution of injury, and potential for response to therapy. These biomarkers would help to differentiate infants who do not require treatment from those at risk of permanent sequelae; infants that might benefit from intervention from those for whom treatment is futile and identify infants who are within a therapeutic window for a specific treatment. It is unlikely that a single biochemical or imaging biomarker measured at a single time point will achieve all these goals. Magnetic resonance imaging (MRI) and spectroscopy (MRS) have shown promise, but the most predictive protocols and the optimal timing of studies is still not fully established [118].

Measurement of inflammatory proteins in blood, including cytokines, shortly after birth in preterm infants may provide information about the risk of sonographic WMI (which correlates with neurodevelopmental outcome). Serial measurements of blood proteins during the first 2 postnatal weeks in extremely low gestational age newborns (born before the 28th week of gestation) in the ELGAN study demonstrated an increased risk of ventriculomegaly, sonographic indicator of diffuse cerebral WMI, in association with elevated concentrations of vascular endothelial growth factor receptor 1, serum amyloid A, and macrophage inflammatory protein 1*β* on day 1 and IL-8 on day 7 [119]. An increased risk of an echolucent lesion, a sonographic indicator of focal cerebral white matter damage, was associated with elevated concentrations of macrophage inflammatory protein 1*β* on day 1 and intercellular adhesion molecule 1 on day 7 [119]. Interestingly, in this same study, elevated concentrations of the chemokine Regulated upon Activation, Normal T-cell Expressed, and Secreted (RANTES, also known as CCL5) was associated with reduced risk of both ventriculomegaly and echolucent lesions. RANTES downregulates TLR4 ligation-induced IL-6 and TNF-*α* secretion by enhancing IL-10 production in PBMCs [120] and may play an anti-inflammatory role in perinatal brain injury.

Elevated cytokine levels have been associated with perinatal brain injury and show promise as diagnostic and/or prognostic biomarkers to be used in a multimodal approach along with MRI. Elevated levels of IL-1*β*, IL-6, IL-8, and lower levels of IL-12 following term delivery in infants with neonatal encephalopathy has been associated with impaired cerebral oxidative metabolism based on MRS and abnormal neurodevelopmental at 30 month of age, but not with detectable MRI changes in the neonatal period [108]. Procianoy and Silveira reported on the association between high cytokine concentrations with WMI in preterm infants and sepsis, looking at cohort of 84 very-low-birth-weight infants, 27 (32%) with WMI, and 57 (68%) control subjects (no WMI). WMI was increased in infants with clinical early-onset sepsis and higher plasma levels of IL-8, IL-6, and TNF-*α*. IL-8 levels ≥100 pg/mL had sensitivity 96%, specificity 83%, and negative predictive value 98% indicating that this chemokine may be a good predictor of WMI [86]. Although elevated levels of CSF cytokines have been associated with WMI, plasma cytokine concentrations may not reflect CSF cytokine levels or inflammatory events within the brain [94]. Therefore, relying on plasma cytokines as biomarkers of perinatal brain injury may prevent early recognition of localized brain inflammation. Additionally, measuring cytokines to assess perinatal brain injury has not been done routinely in the NICU setting and will likely require lowercost, automated, on-demand testing before these potential biomarkers are incorporated into standard diagnostic testing. Multiple assessments of these values over time may provide more accurate predictive values.

## 13. Prevention and Treatment of Perinatal Brain Injury

There are few interventions currently available to prevent or treat perinatal brain injury. Currently used strategies known to improve the outcome of prematurity include maternal prenatal treatments with magnesium sulfate and betamethasone, and postnatal neonatal use of caffeine. The only proven therapy available for term and near term infants with neonatal encephalopathy is therapeutic hypothermia. There are other promising therapies under active investigation for prevention and treatment of neonatal brain injury, including melatonin, erythropoietin (Epo), N-acetylcysteine, Epo mimetics, allopurinol, and xenon. Some of these approaches target anti-inflammatory mechanisms, and still others improve BBB function, thereby preventing the passage of cytokines and other potentially injurious factors into the brain. Examples of such approaches are explored below.

### 13.1. Erythropoietin

 Epo is a hemopoietic growth factor produced by all vertebrates. Functional receptors for Epo are present on cell types other than erythrocyte progenitors, including neurons, and many glial cell types. Epo is a promising novel neuroprotective agent. It is widely available, affordable, and has been safe in over 25 years of neonatal studies of erythropoiesis. Epo triggers several different signaling pathways after binding to its receptor. Neuroprotective effects are associated with activation of Janus kinase/Stat5 and NFkB pathways [121], while Stat5 and Akt pathways are required for neurotrophic effects of Epo [122]. Epo also stimulates expression of several growth factors, including vascular endothelial growth factor secretion (VEGF) [123] and brain-derived neurotrophic factor (BDNF) [124], which may be beneficial in the injured brain. There are extensive data to support its neuroprotective effects *in vitro*, and in neonatal models of brain injury [125–131]. Epo has anti-apoptotic [128, 129] and anti-inflammatory effects (decreased Il-6 and IL-8) [132, 133], and it also increases neurogenesis, [134, 135] and protects oligodendrocytes from injury [136]. These combined effects might provide neuroprotective benefit for brain injury typical of preterm infants and term infants with hypoxic-ischemic injury. Phase I/II studies to determine safety and pharmacokinetics have been done [137, 138], and further phase II/III studies are underway or in the planning stages.

### 13.2. Melatonin

 Melatonin (N-acetyl-5-methoxytryptamine) is a naturally occurring hormone which regulates circadian rhythms. Melatonin has antioxidant [139] and antiapoptotic effects [140, 141]. Prenatally administered low-dose melatonin can reduce cerebral inflammation and apoptosis following birth asphyxia in the spiny mouse [142]. In a fetal sheep model of perinatal asphyxia, melatonin attenuates the production of 8-isoprostanes and reduces activated microglia cells and TUNEL-positive cells in the brain [143]. In a neonatal rodent model of LPS-induced hypoxic-ischemic injury, multiple low-dose treatments with melatonin reduced injury by 45%, but higher dose treatment was not protective [144]. Clinically, melatonin has shown beneficial effects when given to both asphyxiated [145] and septic children [146].

### 13.3. Curcumin

 Curcumin, the main active ingredient in turmeric, can prevent the onset of inflammation by inhibiting activation of NF*κ*B, production of TNF-*α*, IFN-*γ*, and NO, expression of iNOS, and activation of nicotinamide adenine dinucleotide phosphate-oxidase (NOX) [147, 148]. Curcumin has been demonstrated to have a protective effect associated with suppression of iNOS and NOX activation injury in a neonatal rat model of LPS-induced WMI [149].

## 14. Targeting the BBB to Fight Disease

Another approach to preventing or treating neonatal brain injury might be to target the BBB. Several neonatal pathologies involve increased leakiness or dysfunction of the BBB. Therefore, using agents that improve BBB function might improve outcomes. Steroids, hypothermia, intracellular cyclic AMP, adrenomedullin, and noradrenergic agents all stimulate an increase in BBB function. These approaches are under investigation or used therapeutically to treat some adult brain disorders. For example, dexamethasone treatment is currently used to decrease the brain edema associated with brain tumors [150], and Ca^2+^ channel blockers are under investigation as treatment for hypoxia-induced brain injury [151, 152]. Hypothermia, which also improves BBB function, is one of the few proven therapies available to treat neonates with hypoxic-ischemic brain injury and has the lowest number needed to treat to see benefit [153]. Stabilizing activated mast cells with disodium cromoglycate (Cromolyn) may decrease BBB leakiness by inhibiting release of potentially toxic factors including histamine, serotonin, neutral proteases, cytokines, chemokines, and free radicals [154, 155].

Another approach under investigation in adult models of disease is to improve the health of the endothelial cells involved in maintenance of the BBB. The use of exercise, fish oils, and specific fruits, soy, vitamins C an E, and red wine may all be of benefit (NNT = 7–9) [25]. The application of a select group of these strategies might be applicable to neonatal brain injury; however, each one must be studied with regard to safety, efficacy, and developmental implications.

## 15. Conclusion

Large knowledge gaps exist regarding the detailed roles of cytokines in brain injury, repair, and protection in the human fetus/neonate. Although animal studies have demonstrated an important role of cytokines in brain injury, many questions on the underlying cytokine-related mechanisms influencing brain injury remain unanswered. In humans, the fetal/neonatal brain injury knowledge gap is even wider (Table 1), with developmental differences in immune response and in the complex neurovascular barrier mechanisms that play a critical role in regulating inflammatory mediator traffic at the interface between the systemic circulation and the brain. Understanding the balance between pro- and anti-inflammatory mediators and their roles in normal brain development and in the setting of inflammation is needed to tailor treatments that promote neuroprotection.

Future large animal studies aimed at developing diagnostic cytokine profiles of perinatal brain injury biomarkers must be designed to allow evaluation in the context that is clinically useful. While neonatal rodents models of brain injury provide vital information about mechanisms of brain injury and also neuroprotection, it is essential that information learned in these models be verified in larger animal models (fetal sheep, piglet, and nonhuman primate) that more closely reflect human brain development.

For example, for early-hospital diagnosis, a test that is reasonably specific and very sensitive to early perinatal brain injury secondary to infection or cytokines/inflammation would be necessary to facilitate time-sensitive anti-inflammatory strategies. Such a study should be specifically designed to address the incremental benefits of biomarker-based information beyond traditional means of assessment, such as standardized clinical examination, maternal history, risk factor assessment, and radiographic studies. For purposes of identifying risk of early deterioration, additional data might be obtained by serial measurements in the early hospital setting. Similarly, for functional prognosis, serial testing in the subacute setting might provide useful information. Patient heterogeneity (e.g., genetic factors), and the timing, type, degree, and duration of perinatal brain exposure to inflammatory mediators/cytokines likely influence long-term neurodevelopmental outcomes. The need for accurate biomarkers is well illustrated by infants affected by neonatal encephalopathy secondary to hypoxic ischemic encephalopathy. Over 1500 neonates have now been enrolled in randomized controlled trials of therapeutic hypothermia using the *best available entry criteria*: a combination of clinical assessments (Apgar scores, Sarnat or Thompson scores), laboratory assessment (lactic acid, pH, base deficit) and electrophysiologic function [153]. While these criteria identify a group of high risk neonates, their predictive value is poor: untreated, one-third of these infants do well with no long-term neurodevelopmental sequelae, while two thirds die or have significant long-term neurodevelopmental impairment. Treatment improves outcomes by approximately 15%, but the infants who will benefit cannot currently be differentiated from those who will not, nor from those who will do well without treatment.

Similarly, it is unlikely that one single biomarker, such as a cytokine, will be robust enough to have clinical utility for guiding treatment of infants with perinatal brain injury. A panel of biomarkers will therefore likely be more useful. Ideally, future biomarker biomarkers, which incorporate serum cytokine levels and imaging modalities will allow for early tailored individualized treatment strategies that will promote the proper treatment for the proper patient at the proper time. Similarly, in the subacute setting, a biomarker panel might be useful adjunctive tool combined with clinical information and radiographic imaging to determine risk stratification to direct aggressiveness of care for primary or secondary prevention of perinatal brain injury in patients with known risk factors.

## Figures and Tables

**Table 1:** Gaps in knowledge regarding human perinatal brain injury.

Barriers to Accessing the Brain	
(i) Do pathogens, inflammatory mediators and inflammatory cells access the fetal and neonatal brain using the same mechanisms as in animal and adult models?	

Infection	
(i) Which leukocyte populations and which specific proinflammatory cytokines are the primary triggers for brain damage of premature infants?	
(ii) What is the origin and the role of proteins differentially expressed in amniotic fluid associated with chronic chorioamnionitis cases compared to acute chorioamnionitis in the amniotic fluid detected by proteomic analysis?	
(iii) What is the role of microglial IL-1*β* signaling and TIMP expression in perinatal brain inflammation and repair?	
(iv) What are the mechanisms of brain injury from LPS-activated microglia leading to PVL?	

Cerebral Palsy	
(i) What are the roles of inflammatory cytokines in preterm infants that develop CP?	
(ii) To what extent does an altered inflammatory response and persistent neuroinflammation originating in the perinatal period play a long-term role in preterm children with PVL-induced CP?	

Dual Role of Cytokines	
(i) What variables determine neuroprotective and neuroinflammatory properties of cytokines (e.g., timing, type, location, and duration of injury)?	

Cytokines and Genetic Susceptibility to Perinatal Brain Injury	
(i) Which cytokine gene polymorphisms predispose to CP?	
(ii) How do cytokine gene polymorphisms interact with perinatal infections to cause CP?	

Cytokine Biomarkers of Perinatal Brain Injury	
(i) Are there accurate diagnostic, predictive, and prognostic cord blood and neonatal plasma cytokines biomarkers that reflect CSF cytokine levels or inflammatory events within the brain?	
(ii) Are there biomarkers specific for precise inflammatory conditions associated with white matter injury (e.g., differentiating between septicemia and necrotizing enterocolitis) that will provide time-sensitive, pathogen and treatment specific information?	

Prevention and Treatment of Perinatal Brain Injury	
(i) Which anti-inflammatory cytokines and treatments will safely and effectively alter cytokine profiles promoting neuroprotection and repair?	
(ii) What is the optimal timing of such treatments?	

Abbreviations: TIMP: tissue inhibitors of metalloproteinases, LPS: lipopolysaccharide, PVL: periventricular leukomalacia, CP: cerebral palsy, CSF: cerebrospinal fluid.
